# Dendritic Cell Apoptosis and the Pathogenesis of Dengue

**DOI:** 10.3390/v4112736

**Published:** 2012-11-01

**Authors:** Sharon de T. Martins, Guilherme F. Silveira, Lysangela R. Alves, Claudia Nunes Duarte dos Santos, Juliano Bordignon

**Affiliations:** 1 Laboratory of Molecular Virology of the Carlos Chagas Institute, ICC/Fiocruz, Prof. Algacyr Munhoz Mader Street 3775, CIC, Curitiba, Paraná, 81350-010, Brazil; Email: sth_martins@yahoo.com.br (S.T.M.); gfsilveira@gmail.com (G.F.S.); 2 Laboratory of Gene Expression Regulation of the Carlos Chagas Institute, ICC/Fiocruz, Prof. Algacyr Munhoz Mader Street 3775, CIC, Curitiba, Paraná, 81350-010, Brazil; Email: lys.alves@tecpar.br

**Keywords:** Dendritic cell, apoptosis, dengue virus

## Abstract

Dengue viruses and other members of the *Flaviviridae* family are emerging human pathogens. Dengue is transmitted to humans by *Aedes aegypti* female mosquitoes. Following infection through the bite, cells of the hematopoietic lineage, like dendritic cells, are the first targets of dengue virus infection. Dendritic cells (DCs) are key antigen presenting cells, sensing pathogens, processing and presenting the antigens to T lymphocytes, and triggering an adaptive immune response. Infection of DCs by dengue virus may induce apoptosis, impairing their ability to present antigens to T cells, and thereby contributing to dengue pathogenesis. This review focuses on general mechanisms by which dengue virus triggers apoptosis, and possible influence of DC-apoptosis on dengue disease severity.

## 1. Dengue Virus

**Figure 1:**
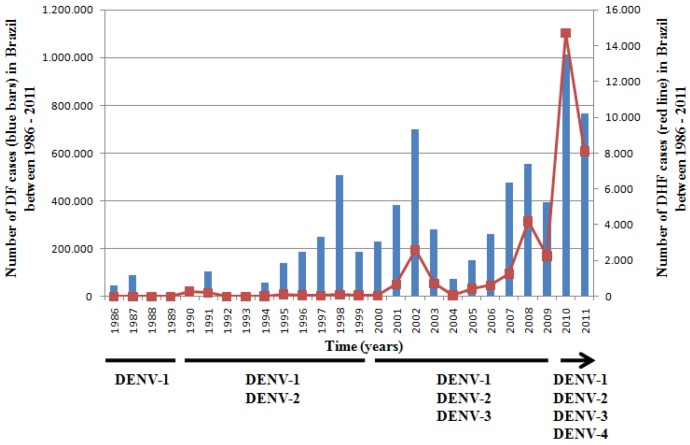
Incidence of dengue fever (blue bars) and dengue hemorrhagic fever (red line) in Brazil since its re-introduction in 1986.

Dengue virus (DENV) is the most important arboviral infection in the tropical and sub-tropical regions of the world affecting more than 100 countries [1]. About two fifths of the world’s population lives in areas where there is a risk of dengue infection, and dengue affects 50 million people annually causing more than 25,000 deaths [2]. In Brazil, after a period of more than 30 years without DENV circulation, dengue virus serotype-1 (DENV-1) was detected in 1981 in the North region of the country [3] and caused a limited epidemic. In 1986, DENV-1 was re-introduced into the country through the Rio de Janeiro port, and since then more than 6 million cases have been confirmed with almost 2000 deaths up to December 2011 (Figure 1). All four dengue virus serotypes currently circulate in Brazil (hyperendemicity) and in the last 10 years the country has experienced an increase of more than 20-fold in DHF cases and deaths by dengue infection [4,5].

DENV belongs to the *Flaviviridae* familiy, genus *flavivirus.* The dengue virus is an icosaedrical particle, with approximately 50 nm, and contains a positive strand RNA genome of nearly 11Kb. The Dengue genome codes for three structural proteins (C, prM/M and E) and seven non-structural proteins (NS1, NS2A, NS2B, NS3, NS4A, NS4B and NS5) responsible for the structure and organization of replication complex, and of the virus particle, respectively [6,7].

Infection with any of the four DENV-serotypes can cause severe or non-severe Dengue. In severe clinical presentations can occur dengue hemorrhagic fever (DHF) and dengue shock syndrome (DSS) [8,9]. Non severe Dengue (Dengue fever) was further classified in “dengue with warning signs”. Patients that present Dengue with warning signs need to be hospitalized, since they will probably develop severe forms of disease. Patients that present Dengue without warning signs can be sent home [8]. Dengue fever (DF) usually involves high fever (≥40 °C for 2–7 days), generally accompanied by severe headache, retro-orbital pain, muscle/joint pains, nausea, vomiting, swollen glands and/or rash [9]. Cases of dengue hemorrhagic fever (DHF) and dengue shock syndrome (DSS) present symptoms of DF and also severe abdominal pain, rapid breathing, fatigue, restlessness and low blood pressure in DSS [9]. The main pathological finding in DHF/DSS is plasma leakage due to endothelial damage during the infection (for a review see [10]).

There have been several hypotheses to explain why DHF/DSS occurs more frequently, but not exclusively, in heterologous secondary infections. Dengue hemorrhagic fever may be triggered due to antibody-dependent enhancement [11] and original antigenic sin of T cells [12]. Also, differences between viral strains [13,14] may also contribute to dengue pathogenesis, and indeed, it has been demonstrated that a DENV-strain isolated from a fatal dengue case induce higher apoptosis rates in dendritic cells than a strain isolated from a non-fatal case [15]. The host response to DENV [16,17] also accounts to DHF/DSS pathogenesis, as demonstrated for the type I IFN response after DENV infection [17]. Recently, host genetic polymorphisms of several genes, notably TNF-α [18], TAP [19], and the DC-SIGN promoter region [20] have been related to DHF/DSS. Finally, ethnic factors [21] and age [22] have been also correlated with DFH/DSS, albeit to a lesser extent. These various putative mechanisms are not mutually exclusive, and the combination of ADE / T-cell antigenic sin / viral strain / individual background / nutritional status may favor high viremia and the cytokine storm observed in DHF/DSS [11,12,13,14,18,19,20,21,22,23].

The host response to DENV infection starts with dendritic cells at the dermis, since resident dendritic cells and Langerhans cells in the dermis are the first cell targets of DENV infection [24]. Dendritic cells are specialized cells that can process and present antigens to T lymphocytes, and are therefore responsible for the induction of adaptive immune responses [25]. Interactions between DENV and DCs have a crucial role in the control of DENV infection, either directly or due to the stimulation of dengue-specific T lymphocytes, and may contribute to determining whether or not DHF/DSS develops.

## 2. Dendritic Cells

Dendritic cells are amongst the most important antigen presenting cells in humans and other mammals. Ralph M. Steinman and Zanvil Cohn first described these cells in 1973, as phagocytic cells with dendrite-like protrusions [26]. Monocytes, macrophages and dendritic cells have a common and exclusive precursor, the macrophage-DC progenitor (MDP), which differentiates into the common DC progenitor (CDP) generating precursor DCs (pre-DCs). Pre-DCs can migrate to lymph nodes, proliferate and differentiate into DCs [27]. Monocytes are derived from common myeloid progenitors (CMPs) and MDPs, and are known as classical macrophage precursors. Cells derived from pre-DCs were first classified as conventional DCs; they display classic DC form and function in steady state conditions. Conventional DCs comprise migratory DCs and lymphoid DCs. Migratory DCs are tissue-resident, and once they find an antigen, these cells migrate to lymph nodes, where they stimulate T-Lymphocytes. Lymphoid DCs are restricted to lymphoid tissues and are generally classified according to the expression of CD4 or CD8 (reviewed by [28]). Monocyte-derived DCs (CD11c+) and plasmocytoid DCs (CD123+) are referred as non-conventional DCs [28,29]. Particular DC subsets can be generated from monocytes during inflammation, although they have been reported even in steady-state conditions. Monocyte-derived DCs are classified as non-conventional mainly because of their origin and are commonly found in the periphery, migrating to draining lymph nodes whenever they find an antigen (reviewed by [28]). Monocyte differentiation into DCs can enhance antiviral immunity, and it was recently demonstrated that viral infection can induce monocyte differentiation into a CD16(–) CD83(+) DC subset with a strong potential to activate T cells [30]. Plasmocytoid DCs are found in lymphoid and non-lymphoid tissues, and express two types of Toll-like receptors (TLR7 and TLR9) that mediate the expression of interferon regulatory factor 7 (IRF7), a transcriptional activator that can modulate the production of type I interferon [31]. The secretion of IL-6 and IFN type I promotes the differentiation of activated B cells in plasma cells, highlighting the importance of this cell type in immune response modulation [32]. Plasmocytoid DCs also appear to be involved both in induction of tolerance and in modulation of autoimmune responses [33].

Dendritic cells have different activities and functions depending on their maturation status. Immature DCs (iDCs) are not only precursors, but also potent phagocytes that can capture and process antigens that may then be used to form MHC-peptide complexes (reviewed by [34]). Immature DCs express low levels of MHC-I, MHC-II and co-stimulatory molecules (CD80, CD83 and CD86) on their cell surface. As a consequence of this low expression, iDCs have a limited ability to present antigens. They can patrol peripheral areas and migrate to infection sites, coordinated by inflammatory chemokines. This explains the expression of chemokine receptors, like CCR1, CCR2, CCR5, CCR6, CXCR1, and CXCR2 on the surface of iDCs [35,36,37]. 

Maturation stimuli cause a decline on antigen uptake rates, as shown for some TLR ligands like Poly I:C and LPS [38]. An immunoproteasome is formed, and antigen processing in the now mature cells is facilitated by the acidification of endosomal compartments, proteosomal alterations and cathepsin activation [39,40]. Late events in the maturation process are the upregulation of genes related to antigen presentation [37] and down-regulation of receptors that recognize pro-inflammatory cytokines [35]. To interact with T-cells in the lymphoid tissues, the expression of several proteins in DCs is regulated. The half-life of MHC-I complexes on the cell surface increases, and activation molecules like MHC-I, MCH-II, and co-stimulatory factors like CD80, CD83 and CD86 are overexpressed (reviewed by [34]).

## 3. Dendritic Cells and Dengue Virus

Development of DHF or DSS may be related to the ability of DCs to counteract dengue replication [41]. Following DENV inoculation in the dermis the primary targets for infection are skin-resident Dendritic Cells (DCs) and Langerhan’s Cells (LCs) [24,42]. The specific receptor for DENV binding in DCs is the dendritic cell-specific intercellular adhesion molecule 3 grabbing non-integrin (DC-SIGN or CD209) [43]. DCs additionally sense pathogens by various pathways, involving Toll-like receptors (TLR), RIG-like receptors (RLR) and NOD-like receptors (NLR) (reviewed by [44]). 

DENV-infected DCs acquire a maturation profile, expressing characteristic surface molecules like CD40, CD80, CD83, and CD86 [45,46]. These surface proteins facilitate a more efficient interaction between DENV-infected DCs and T-cells [47], and also contribute to B cell activation and differentiation [48,49,50]. Uninfected DENV-exposed DCs are also activated following the recognition of defective virus particles or proteins, the action of DC-secreted exosomes [51] and/or the effects of cytokines secreted by infected DCs (TNF-α, IFN type I and II ) [52]. DENV-infected DCs start the production of IL-6 [53], and upon activation, uninfected DENV-exposed DCs and infected DCs migrate to the lymph nodes and prime T cells through the major histocompatibility complex (MHC) type I and II, initiating the adaptive immune responses [54]. DENV infected DCs have increased expression of TNF-α, programmed death ligand 2 (PD-L2) and MHC-II, and a lower expression of IFN inducible protein 10 (IP-10), programmed death ligand 1 (PD-L1), CD80, CD86 and MHC-I, when compared with bystander DCs [55].

Pro-inflammatory cytokines, like TNF-α and IFN-α secreted by DENV-infected DCs enhance the activation of DCs and other immune cells, induce apoptosis of endothelial cells and the up-regulation of interferon-stimulated genes (ISGs) with antiviral activity [45,53,56]. Other important cytokines secreted by DENV-infected DCs are IL-6, IL-8 [15] and IL-12p70 [45]. Chemokine production by DENV-infected DC, including RANTES, CXCL9, MCP-1, IP-10, and IL-8, has also been demonstrated [23] and may contribute substantially to the plasma leakage and local inflammation, with cells overflowing into tissues. However, non-structural flavivirus proteins, notably NS5 [57], NS4B [58] and the NS2B-NS3 complex [59], are able to inhibit type I IFN responses compromising the innate immune response. This mechanism could be used to weaken the antiviral defense mechanisms, although it has not been demonstrated yet for DENV- infected dendritic cells. 

Dendritic cells infected with dengue virus may also contribute to the development of vascular leakage through the secretion of metalloproteinases [60]. It has been shown that soluble gelatinolytic matrix metalloproteinase (MMP)-9 and MMP-2 enhance endothelial permeability by reducing the expression of the endothelial adhesion molecule 1 (PECAM-1) and vascular endothelium (VE)-cadherin cell adhesion molecules, and by causing a redistribution of F-actin fibers [60].

## 4. Apoptosis

Type I programmed cell death or apoptosis is a controlled mechanism that is triggered under normal physiological conditions, for example during development and aging, as well as in response to various stresses and pathologies, including viral infection [61,62]. There are two main apoptotic pathways that are interconnected: the extrinsic pathway and the intrinsic (autonomous) pathway. The extrinsic pathway is mediated by transmembrane receptor-mediated interactions, called death-receptors. The best characterized are the Tumor Necrosis Factor (TNF) and the Fas receptors [63,64]. Once these receptors are activated they lead to the formation of the death-inducing signaling complex (DISC), activation of caspases 8 and 10 and the beginning of the execution phase of apoptosis. Members of the Bcl-2 protein family, which can act either as pro-apoptotic or anti-apoptotic factors, regulate the intrinsic pathway. The pro-apoptotic Bax and Bak transmembrane proteins form pores in the mitochondrial membrane allowing the release of cytochrome C, and the formation of apoptosomes; this leads to the activation of caspase 9, that activates caspase 3 resulting in the start of the execution phase as well as triggering the extrinsic pathway [61,62]. During the execution phase, caspase 3 activates CAD protein, an endonuclease that degrades the chromosomal DNA and also induces cytoskeleton collapse leading to the formation of apoptotic bodies [61,62,65,66]. 

Infection by virus and other pathogens can induce DC apoptosis. During measles virus infection, apoptosis is induced by Fas- and TRAIL-mediated pathways [67]; in foot and mouth disease, apoptosis occurs through the binding of viral proteins to integrin receptors [68] and DENV-induced cytokines seems to trigger DC apoptosis [53]. DC homeostasis also has an important role in tolerance induction, as DC apoptosis induces the formation of antigen-specific Tregs through engulfment of apoptotic DCs by viable DCs [69]. Tregs in this microenvironment also have increased activity [70] and negatively modulate the activity of the remaining DCs [71].

## 5. Dengue Virus-Induced Apoptosis

Experimental and clinical data suggests that DENV has a wide range of cellular and tissue tropisms. DENV antigens have been found in mononuclear cells, B cells, Langerhans cells in the skin, mouse neurons, human endothelial cells, liver cells, heart and skeletal muscle ([24,25,72,73,74,75,76] for a review see [77]). Apoptosis induction by DENV in a broad of cell types can contribute to dengue pathogenesis. Autopsy examinations of fatal DHF/DSS cases have demonstrated apoptotic cells in liver, brain, intestinal and lung tissues [78]. Apoptotic microvascular endothelial cells in intestinal and pulmonary tissues may explain the plasma leakage manifestations observed in patients experiencing the severe forms of the disease [78].

The mechanisms by which DENV induces apoptosis are not completely understood and may differ between cell types or tissues [79]. *In vitro* studies have demonstrated that accumulation of DENV-proteins in the endoplasmic reticulum (ER) induces apoptosis of hepatoma cells (HepG2) [80]. Accumulation of viral proteins in the ER membranes, rather than virus release, may cause ER stress and thereby activate the apoptotic pathway, as occurs in mouse neuroblastoma cells [81]. Apoptosis of hepatic cells due to DENV infection may explain the high levels of transaminases and liver damage observed in some DENV patients [82,83,84]. Additionally, NF-κB is activated in DENV-infected hepatocytes [80] and induces the expression of CD137, a member of TNF-α receptor family [85]. Anti-CD137 antibody binding to CD137 may activate caspase cascades triggering apoptosis of hepatocytes [85]. Activation of NF-κB is controlled by its ligation to Daxx, a death domain-associated protein [86]. During DENV infection, capsid protein binds to Daxx [86] and free NF-κB is then available to regulate CD137 expression [87], what could trigger apoptosis of infected cells. Despite its role in the induction of apoptosis, DENV-capsid protein may also contribute to subvert apoptosis: DENV-C protein interacts with the calcium modulating cyclophilin-binding ligand (CMAL), a regulator of intracellular calcium levels [88]. DENV-infected cells express high levels of CAML and consequently have high cytosolic calcium concentration, and this may help DENV to subvert apoptosis since it protects cells against mitochondrial damage [89]. 

In neuroblastoma cells infected with DENV, apoptosis seems to be induced due to production of phospholipase A2 (PLA2). This enzyme converts membrane phospholipids to arachidonic acid (AA), a major lipid second mediator of several intracellular reactions [90]. PLA2 activation is essential for the induction of apoptosis, and occurs in response to diverse stress stimuli, such as TNF-α and FasL signaling [91]. DENV infection induces the activation of PLA2 and production of AA [92], and PLA2 is abundant in the serum of DENV-infected patients [93]. Arachidonic acid stimulates the synthesis of NADPH oxidase, producing superoxide anions and other reactive oxygen species (ROS), and these are detected in neural cells infected with DENV-2 [94]. ROS can function as signal transducers and activate molecules like NF-κB that regulates the expression of several genes involved in immune responses [95]. The well-studied tumor suppressor p53 has its expression regulated by NF-κB and is involved in apoptosis and cell cycle arrest. SK-N-SK cells infected with DENV-2 express both NF-κB and p53, a further evidence for the role of those molecules in DENV-induced apoptosis [94]. Other dengue virus proteins, like prM/M [96], envelope (E) and helicase NS3 [97], participate in the control of apoptosis (for a review see [79]).

The apoptosis of endothelial cells, one of the major findings in DHF/DSS, may be induced directly by viral replication in infected cells or by DENV-NS1 activating the complement system [98,99]. DENV-NS1 is secreted from infected mammalian cells as soluble hexamers (sNS1) [100] but can also remain associated with membranes of infected cells as dimers [101]; free sNS1 itself, or in the presence of anti-sNS1 antibodies, can directly activate the complement system. In the presence of specific antibodies, complement may be activated even by cell-associated NS1 [99]. However, it was recently demonstrated that the sNS1 from DENV, West Nile and Yellow Fever Virus interacts with C4 and attenuates complement activation [102].

The mechanism of the apoptosis induced by DENV also appears to depend on the viral strain and/or serotype. Receptor-interacting serine/threonine protein kinase 2 (RIPK2) is a mediator that activates caspases, NF-κB and other kinases. RIPK2 contributes to DENV-mediated apoptosis induced by capsid protein but only for infections involving DENV-2 and -4 serotypes [103]. Additionally, it was recently demonstrated that DENV-3 isolated from a fatal case induces higher rates of apoptosis in monocyte-derived DCs than a strain isolated from a non-fatal case of DF [15].

## 6. Dendritic Cells Apoptosis: Mechanisms and Dengue Pathogenesis

Dendritic cell apoptosis has a role on immune regulation, because it controls antigen availability to T cells and any alteration in DC cell death has a major effect on the antigen-specific immune response, inflammation and immune tolerance [104]. In the absence of feeding citokynes (GM-CSF and IL-4), DCs undergo spontaneous apoptosis [53]. IL-10 induces cell death by down-regulating anti-apoptotic proteins such Bcl-2 and TGF-β [105,106,107,108]. 

The mechanisms by which some members of the *Flaviviridae* family induce apoptosis in DCs have been established. HCV proteins NS3, NS4, polyprotein NS3-NS4, NS5 and core protein induce DCs apoptosis through FasL expression. As a consequence, important antiviral signaling, such as Toll-like pathway, NF-κB, and secretion of inflammatory mediators, like IL-12 are inhibited [109]. Type I IFN is important for antiviral responses, but high type I IFN secretion during infections with HBV and HCV can contribute to DC apoptosis via the intrinsic pathway [110]. Classical Swine Fever Virus (CSFW) infection induces activation of plasmocytoid and conventional DCs [111] and the virus can replicate inside DCs, causing apoptosis and controlling type I IFN responses [112]. The cell death mechanisms induced by DENV in other cell types, and particular features of DC apoptosis induced by members of *Flaviviridae* family, implicate Ca^2+^, PLA2, AA, ROS, cytokines and other mediators in the apoptotic process. However, further studies are necessary to establish the exact pathways involved in DENV-mediated DC apoptosis, once different DENV serotypes and strains may induce DC apoptosis through different mechanisms.

**Figure 2:**
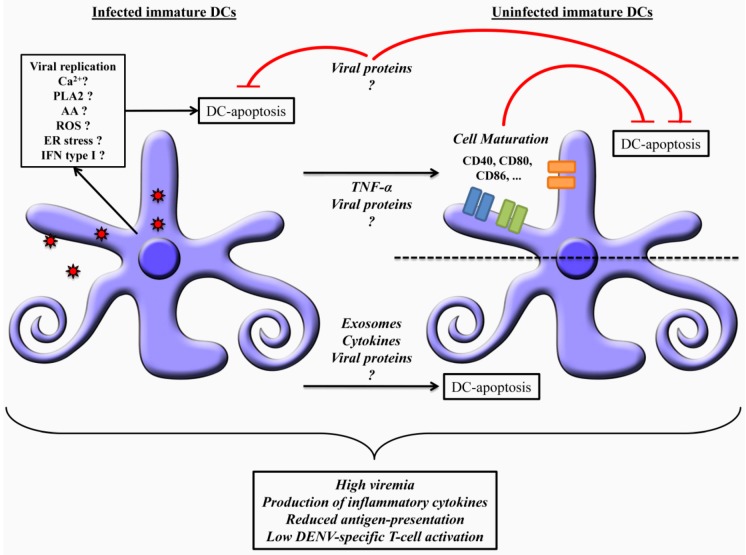
Possible mechanisms by which dengue virus may induce apoptosis of Dendritic cells (DCs). Dengue virus (DENV) can directly induce DC-apoptosis through replication inside infected cells. Apoptosis may also be induced in infected and uninfected-DC via exosomes, cytokines and viral proteins secreted from infected DCs. It has been demonstrated that TNF-α protects DCs from apoptosis induced by DENV, possibly inducing DC maturation. Also, structural and non-structural viral proteins may either induce apoptosis or protect DC from apoptosis. Finally, induction of apoptosis by DENV in infected and uninfected DC presumably contributes to dengue pathogenesis by promoting high viremia, production of inflammatory cytokines, reduced antigen-presentation and low DENV-specific T-cell activation.

Despite the specific pathway used by DENV to induce DC apoptosis, this phenomenon certainly have consequences for dengue pathogenesis. It is therefore plausible that apoptosis of DCs (directly and indirectly) enhances Dengue viremia and cytokine production favoring the development of severe forms of Dengue disease (Figure 2). Also, apoptosis may facilitate viral escape, contributing to tissue damage and pathogenesis, as occurs during infection with some filovirus, picornavirus, and flavivirus [113].

Recently, it was demonstrated that the plasmacytoid and myeloid dendritic cell counts were reduced in the blood of Dengue patients. However, no differences in these counts were observed between mild and severe cases [114]. Furthermore, the numbers of myeloid and plasmacitoyd DCs were significantly lower in DENV patients with high viremia than in non-infected controls, suggesting a correlation between viremia and DC counts [114]. Several explanations can be proposed for the low DC counts in dengue-infected patients: (1) viral cytopathic effects; (2) death of DC induced by ADE; (3) DC migration to secondary lymphoid organs and 4) virus-driven impairment of bone marrow function leading to lower DC production [114].

In addition to its effects on infected cells, DENV can also kill uninfected neighbor cells (bystander effect), contributing to weakening the host immune response [15,115]. An example of indirect induction of apoptosis is provided by the Ebola virus (EBOV), which induces massive apoptosis of T CD4, T CD8 and NK lymphocytes, although the virus does not directly infect these cells [116]. It has been suggested that DENV-induced DC apoptosis is dependent on viral infection and replication [15,52]. However, it was recently demonstrated that bystander apoptosis of DENV-uninfected DCs is strain dependent and that TNF-α secreted during DENV infection protects DCs from apoptosis [15], possibly by inducting DCs maturation [117]. Some viruses, such as Foot and Mouth Disease Virus (FMDV) can even induce DC apoptosis and affect its functions before the infection, as shown for murine Bone Marrow Derived DCs (BMDCs). The apoptosis of DCs due to FMDV impairs the connection between host innate and adaptive immune responses [68]. Additionally, Measles virus (MV) induces apoptosis of DCs contributing to viral spreading, through the releasing of virus (that have an intense replication on this cells) or the contamination of other phagocytes that engulf apoptotic bodies released by dying cells. The contact of bystander DCs with apoptotic MV-infected DCs can induce cell maturation. DCs can also spread MV to secondary lymphoid organs, where they get in touch with CD40L signals from activated T cells. The contact with CD40L can generate cytotoxic DCs that are unable to prime naïve T cells, and moreover can lead activated T cells to death and inhibit their proliferation. It results in an immune suppression that persists even after MV clearance, since the organism needs a time to recover these cells [118].

Moreover, DC apoptosis induced by dengue virus infection would be a mechanism which contribute to dengue pathogenesis, as already demonstrated for other diseases, like Measles Virus infection [67,118], *Streptococcus pneumoniae* [119,120], Foot and Mouth Disease Virus [68], and Malaria [121]. Additionally, in 2005, Palmer *et al*., demonstrated that DENV-infected DCs secrete IL-10 and were apoptotic [52]. The authors suggest that those results are an immune evasion mechanism used by DENV to escape immune response.Therefore, we believe that DC-apoptosis contributes to pathogenesis of infectious disease due to impairment of innate immune response or as a viral escape from immune surveillance.

## 7. Concluding Remarks

DC-DENV interaction is the first point for the immune control after DENV infection. DCs may reduce DENV replication through secretion of type I IFN. However, some DENV strains are able to replicate more efficiently in these cells, due to mutations in non-structural genes, which consequently inhibit the type I IFN response [52,57,59]. In these cases, DENV replication may induce DC apoptosis directly and may even induce apoptosis of uninfected DCs through the action of cytokines, viral proteins or exosomes secreted by infected DCs. Apoptosis of DENV-infected and uninfected DCs may weaken the host immune response, increasing the viral load and cytokine storm observed in severe dengue cases (see Figure 2). Elucidating the mechanisms that control DC apoptosis after DENV infection would be a significant step forward in the understanding of dengue pathogenesis, and consequently for vaccine and antiviral development. 
